# Continuous administration of a p38α inhibitor during the subacute phase after transient ischemia-induced stroke in the rat promotes dose-dependent functional recovery accompanied by increase in brain BDNF protein level

**DOI:** 10.1371/journal.pone.0233073

**Published:** 2020-12-04

**Authors:** John J. Alam, Michael Krakovsky, Ursula Germann, Aharon Levy

**Affiliations:** 1 EIP Pharma, Inc., Boston, Massachusetts, United States of America; 2 Pharmaseed Ltd., Ness-Ziona, Israel; Universitat zu Lubeck Sektion Naturwissenschaften, GERMANY

## Abstract

There is unmet need for effective stroke therapies. Numerous neuroprotection attempts for acute cerebral ischemia have failed and as a result there is growing interest in developing therapies to promote functional recovery through increasing synaptic plasticity. For this research study, we hypothesized that in addition to its previously reported role in mediating cell death during the acute phase, the alpha isoform of p38 mitogen-activated protein kinase, p38α, may also contribute to interleukin-1β-mediated impairment of functional recovery during the subacute phase after acute ischemic stroke. Accordingly, an oral, brain-penetrant, small molecule p38α inhibitor, neflamapimod, was evaluated as a subacute phase stroke treatment to promote functional recovery. Neflamapimod administration to rats after transient middle cerebral artery occlusion at two dose levels was initiated outside of the previously characterized therapeutic window for neuroprotection of less than 24 hours for p38α inhibitors. Six-week administration of neflamapimod, starting at 48 hours after reperfusion, significantly improved behavioral outcomes assessed by the modified neurological severity score at Week 4 and at Week 6 post stroke in a dose-dependent manner. Neflamapimod demonstrated beneficial effects on additional measures of sensory and motor function. It also resulted in a dose-related increase in brain-derived neurotrophic factor (BDNF) protein levels, a previously reported potential marker of synaptic plasticity that was measured in brain homogenates at sacrifice. Taken together with literature evidence on the role of p38α-dependent suppression by interleukin-1β of BDNF-mediated synaptic plasticity and BDNF production, our findings support a mechanistic model in which inhibition of p38α promotes functional recovery after ischemic stroke by blocking the deleterious effects of interleukin-1β on synaptic plasticity. The dose-related *in vivo* efficacy of neflamapimod offers the possibility of having a therapy for stroke that could be initiated outside the short time window for neuroprotection and for improving recovery after a completed stroke.

## Introduction

Stroke is a frequent cause of death as well as a leading cause of acquired disability worldwide and is associated with a substantial economic burden due to high costs for treatment and post stroke care [1, 2]. Approximately 80% of strokes are ischemic in nature due to thromboembolic occlusion of a major artery or its branches, leading to a cascade of events that causes irreversible tissue injury [3]. Based on pathological characteristics and their timing, a stroke is classified into three clinical phases, including the acute (i.e. first 48 hours after stroke onset), the subacute (from 48 hours to >6 weeks post stroke) and the chronic phase (starts at 3–6 months post stroke) [4, 5]. The acute phase represents an opportunity to salvage threatened tissue and reduce the extent of injury (i.e. provide neuroprotection), for example via reperfusion or neuroprotection while the subacute phase represents the recovery stage [5, 6]. The subacute phase is characterized by brain repair initiation, so therapeutic strategies include enhancing the underlying spontaneous recovery processes, modifying inflammation, lifting diaschisis, or reducing late neuronal death [5, 6]. Only regenerative approaches would generally be considered to be potentially active in the chronic phase [7].

The only approved pharmacological intervention for acute ischemic stroke is intravenous thrombolysis with recombinant tissue plasminogen activator (TPA), resulting in recanalization of occluded vessels if applied within a short time period (up to 4.5 hours) after stroke [8]. Numerous other attempts at providing neuroprotection during the acute phase of stroke have failed [9–11] and there is an urgent need for alternative, more widely applicable treatment options for ischemic stroke. Such therapeutics might enable treatment of patients who present after the very short time window for thrombolysis, and of patients who are ineligible for intravenous TPA treatment. In particular, there is high interest in the development of novel therapies that are directed at promoting functional recovery from stroke via increasing neuronal and synaptic plasticity during the subacute phase [5, 9, 12]. The main goal is to identify disease-modifying treatments that can be administered after the acute phase of stroke is complete, i.e. treatments that can be administered during the subacute and/or chronic phase [5, 9, 12]. It is expected that the proposed approaches generally target compromised cerebral tissue and/or surrounding intact tissue to promote brain plasticity [5, 12].

For a number of reasons the proinflammatory cytokine interleukin-1beta (IL-1β) is considered a therapeutic target for treatment of ischemic stroke to promote recovery after stroke. IL-1β is upregulated after ischemic stroke [13–17] and in subacute/chronic inflammatory conditions, IL-1β is known to be a key component of the inflammatory response in the brain that mediates neurodegenerative effects of inflammation on cognition and synaptic plasticity [18]. Chronic elevation of IL-1β, such as IL-1β elevation in the aging brain, suppresses brain-derived neurotrophic factor (BDNF) production [19, 20], and *in vitro* it has been shown that increased IL-1β inhibits BDNF effects on neuronal/synaptic plasticity via induction of the intracellular kinase, p38 mitogen-activated-protein-kinase (MAPK) [17, 21]. In the context of recovery after stroke, these effects of IL-1β on BDNF may be a significant block to recovery as BDNF plays a critical role in neural plasticity and recovery after stroke [22]. P38 MAPK otherwise is a key component of IL-1β signaling, particularly with respects to its proinflammatory effects. In the brain, IL-1β signals both through the ubiquitous, but low-affinity proinflammmatory IL-1 receptor accessory protein (IL-1RAP) that signals through p38 MAPK, as well through a high-affinity neuron-specific isoform of IL-1RAP that signals via a different kinase, the non-receptor tyrosine kinase Src [23, 24]. IL-1β signaling via the ubiquitous p38 MAPK-dependent IL-1RAP inhibits long-term potentiation, while signaling via the neuron-specific Src-dependent IL-1RAP facilitates long-term potentiation [25]. These two distinct IL-1β signaling pathways, a low-affinity p38 MAPK-dependent one and a high-affinity Src-dependent one, may be the reason why in many systems low basal concentrations of IL-1β promote synaptic plasticity, while at high concentrations (i.e. those seen under inflammatory conditions) IL-1β inhibits synaptic plasticity [17, 20, 25]. All the above-described research findings taken together stimulated the idea to investigate whether inhibition of p38 MAPK with a specific small molecule inhibitor may have beneficial efficacy as a subacute phase stroke treatment via promoting functional recovery through blocking the deleterious effects of IL-1β on BDNF action and production, and with it on synaptic plasticity.

Otherwise, activation of p38 MAPK, particularly the alpha isoform (p38α), after experimental ischemic stroke in rodents has been demonstrated in neurons, astrocytes and microglia [26–30], and p38α has been established as a driver of neuroinflammation-mediated cell death in the acute phase of ischemic stroke [31, 32]. Therefore, several inhibitors of p38 MAPK that exhibit different potency and kinase selectivity, all of them most potently blocking p38α versus other p38 isoforms (p38β, p38γ, p38δ) have been administered during the acute phase in experimental models of cerebral ischemia, and all of them have provided robust neuroprotection [30, 33–37]. Importantly, administration of a p38 MAPK inhibitor was neuroprotective (i.e. reduced infarct size) when administered up to 6- and 12-hours post stroke, but not when administered 24 hours post stroke in a rat transient middle cerebral artery (tMCAO) model [30].

Heretofore, beyond its role during the acute phase, it had been unclear whether p38α also plays a role in impairing functional recovery during the subacute phase of stroke which embodies brain repair initiation [4–6]. Time course analyses of phospho-p38 (i.e. the activated from of p38) expression in experimental rat stroke models show a biphasic response. In association with the acute inflammatory response, marked increases in phospo-p38, including phospho-p38α, are seen immediately after cerebral ischemia within neurons and other cell types [27, 29, 30, 36]. The acute increase primarily resolves within the first 24 hours, but then there is progressive elevation to 10 to 14 days, the last time points measured in the two studies that assessed p38 expression beyond the acute phase [36, 38]. A one-week study of a novel p38α inhibitor, the tetra-substituted thiophene VCP979, evaluated as a treatment of photothrombotic ischemic stroke in streptozocin-induced Type 2 diabetes mellitus (T2DM) mice demonstrated beneficial efficacy with functional outcome seven days post stroke, associated with both neuroprotection (reduced infarct volume) and axonal/white matter remodeling within the motor cortex [37]. Whereas this study offered novel mechanistic insights for a p38α inhibitor that are relevant for brain repair, it did not address whether the functional recovery was due to neuroprotection or due to effects on recovery mechanisms, as VCP979 administration started at 24 hours post stroke [37], at a time point that is within the acute phase of photothrombotic stroke in mice [39, 40]. Indeed, as VCP979 demonstrated almost an approximate two-thirds reduction in infarct size compared to vehicle administration, the improved functional recovery was likely due to its neuroprotective effects [37].

The pyrimido pyridazine neflamapimod (International Non-proprietary Name for the molecule previously code-named VX-745) is a potent, highly selective, ATP-competitive inhibitor of p38α that yields higher central nervous system (CNS) than peripheral blood exposure after oral administration [41, 42]. It exhibits efficacy and safety profiles in preclinical species to merit ongoing clinical investigation for CNS disorder indications [42]. Neflamapimod is currently being evaluated in the clinic for its potential to reverse synaptic dysfunction in three different CNS disorders (Alzheimer's disease, Huntington's disease, dementia with Lewy bodies). Phase 2 clinical studies in Alzheimer’s disease have been reported [42–44].

Neflamapimod has a potency (IC50) between 10 and 15 ng/mL for IL-1β-induced production of IL-6 or IL-8 from human peripheral blood mononuclear cells (i.e. for IL-1β signaling) [45]. Accordingly, neflamapimod was evaluated in 20- to 24-month old Fischer rats with cognitive deficits, which previously have been described to be due to IL-1β-induced impairment of synaptic plasticity [46]. Results showed that neflamapimod administered via oral gavage at a dose level of 1.5 mg/kg administered twice daily reversed deficits in spatial learning in the Morris-Water-Maze test, a measure of synaptic plasticity. However, that same dose had no effects on hippocampal IL-1β levels, while a 3-fold higher dose (4.5 mg/kg administered twice daily) reduced IL-1β levels in the hippocampus but did not result in pro-cognitive effects [45]. These results suggested that the neflamapimod pro-cognitive effects were mediated through decreasing IL-1β signaling in hippocampal neuron target cells, rather than through reducing IL-1β production [45].

In recognition of the unmet need for stroke therapies that enable initiation of therapy at a time point beyond the therapeutic window for neuroprotection and show potential for promoting functional recovery via beneficial effects on synaptic plasticity, the objectives of the present study were (1) to evaluate neflamapimod at two previously reported pharmacologically active and clinically relevant dose levels for its *in vivo* efficacy to promote neurologic recovery as assessed by the modified neurological severity score (mNSS) and additional behavioral tests in a rat tMCAO model; (2) to initiate administration of neflamapimod outside the known neuroprotection window for p38α inhibitors and to address whether p38α inhibitor treatment is still effective when given at a later time post stroke; (3) to assess the neurogenic factor BDNF in the brain as a potential biomarker for monitoring neflamapimod effects on synaptic plasticity; and (4) to measure IL-1β levels as an inflammatory biomarker in the brain after the treatment period to gauge whether chronic inflammation may play a role in this experimental stroke model and whether a neflamapimod effect related to IL-1β signaling may be detectable.

## Materials and methods

To enhance the reproducibility of results presented in this study, a downloadable protocol file has been deposited at http://dx.doi.org/10.17504/protocols.io.bf69jrh6.

### Animals and general health monitoring

Seventy-six young (3-month old) male Sprague Dawley rats (Harlan Laboratories, Israel) weighing 328 g ± 20% were included in the study. The protocol for the study was approved by the Israeli Animal Care and Use Committee (approval number IL-15-01-15) and was conducted in accordance with the Israeli guidelines that conform to the United States Public Health Service's Policy on Humane Care and Use of Laboratory Animals. Animal handling was performed according to guidelines of the National Institute of Health (NIH) and the Association for Assessment and Accreditation of Laboratory Animal Care (AAALAC). To assess the health status of the animals throughout the study, the general health status was monitored daily and body weight was determined weekly.

### Transient middle cerebral artery occlusion

Transient middle cerebral artery occlusion (tMCAO) in the right brain hemisphere was performed on Day 1 according to a modified method of Longa *et al*. [47] as described by Schmid-Elsaesser *et al*. [48], in which a 4 cm length of 4–0 monofilament nylon suture is inserted under anesthesia through the proximal external carotid artery into the internal carotid artery and thence into the circle of Willis, effectively occluding the middle cerebral artery. A thermostatically regulated heating lamp and pad were used to maintain rat temperature at 37°C during the tMCAO procedure. Anesthesia was induced with 4% isoflurane in a mixture of 70% N2O and 30% O2 and maintained with 1.5–2% isoflurane. The surgical wound was subsequently closed, and the animals were returned to their cages to recover from anesthesia. Two hours after occlusion rats were re-anesthetized, monofilament was withdrawn to allow reperfusion, surgical wound was closed, and rats were returned to their cages.

### Neurologic scoring and behavioral evaluations

The individual performing neurologic scoring and behavioral assessments was unaware of the group assignments, thus, performed blinded evaluations. Neurologic scoring by mNSS evaluation according to Chen *et al*. [49] was performed at least one day before the Day 1 tMCAO, and on Day 2, at Week 4, and at Week 6 following tMCAO. The mNSS is a composite of motor, sensory, reflex and balance tests and well-defined score values [49]), that were used to assess the effect of neflamapimod compared to vehicle control. Taking into account the different tests and score values, the mNSS was graded on a composite scale of 0 to 18, in which the normal, healthy animal value was 0 and the maximal deficit value after tMCAO was represented by 18 [49] (S1 Table). An overall mNSS value of ≥10 was predefined as an inclusion criterion for enrolling a rat with stroke into the neflamapimod treatment study.

Additional behavioral evaluations included stepping test, body swing test, and forelimb placement test that were performed before the tMCAO procedure, and at Week 4 and Week 6 following tMCAO.

A stepping test was utilized to assess forelimb akinesia according to Pharmaseed's internal protocol. Each rat was held with its hind limbs fixed in one hand and the forelimb, not to be monitored, in the other, while the unrestrained forepaw touched the table. The number of adjusting steps of the forelimb to be monitored was counted while the animal was moved sideways along the table surface in the forehand and backhand direction over a distance of 85 cm during approximately five seconds. The stepping test was completed for both the left and right forelimbs at all time points indicated above.

For the body swing test, each rat was held approximately one inch from the base of its tail. It was then elevated to an inch above a surface of the table. The rat was held in the vertical axis, defined as no more than 10° to either the left or the right side. A swing was recorded whenever the rat moved its head out of the vertical axis to either side. Before measuring another swing, the rat must have returned to the vertical position. Twenty (20) total swings were counted. A normal rat typically had an equal number of swings to either side. Adjusted body swing scores were calculated as the difference between leftward and rightward swings (i.e. the number of rightward swings subtracted from leftward swings).

For the forelimb-placing test, the examiner held the rat close to a tabletop and scored the rat's ability to place the forelimb on the tabletop in response to whisker, visual, tactile, or proprioceptive stimulation. Separate sub-scores were obtained for each mode of sensory input and added to give total scores (0 = normal, 12 = maximally impaired). Scores were given in half-point increments: whisker placing (0–2); visual placing (forward (0–2) and sideways (0–2)); tactile placing (dorsal (0–2) and lateral (0–2)); proprioceptive placing (0–2).

### Administration of test articles

Test articles, neflamapimod at two different dose levels in 1% (w/v) Pluronic F108 vehicle, or vehicle (1% (w/v) Pluronic F108) were supplied by EIP Pharma, Inc. and administered at a dosing volume of 5 ml/kg by oral gavage twice daily (7 AM and 7 PM) on 5.5 days (i.e. Sunday through Friday at 7 AM and Sunday through Thursday at 7 PM) every week for six weeks (i.e. from Day 3 until Day 42), starting at 48 hours post reperfusion. The dose levels of neflamapimod administered were 1.5 mg/kg and 4.5 mg/kg twice daily.

### Sample collection and analysis

On Day 44, two days following the final dosing of test article or vehicle, rats were sacrificed by CO_2_ inhalation. Brains were harvested from all animals and samples were divided into left and right hemisphere. Samples were weighed, immediately frozen in liquid nitrogen, and stored at -80°C. For the IL-1β and BDNF analyses, tissue samples were defrosted and homogenized in 1 ml/200 mg tissue of 20 mM Tris-HCL pH 7.4 containing a protease inhibitor cocktail (50 μl/ml). Samples were centrifuged at 10,000 x g for 15 minutes at 4°C. Clear supernatants were aliquoted (150 μl/aliquot) and stored at -80°C until enzyme linked immunosorbent assay (ELISA) for IL-1β or BDNF was performed. For IL-1β analysis, ELISA was performed using the Rat IL-1 beta/IL-1F2 Quantikine ELISA Kit (R&D Systems, Minneapolis, MN) according to manufacturer’s instructions. For BDNF analysis, ELISA was performed using the Solid Phase Sandwich Quantikine ELISA Kit (96-well strip plates, R&D Systems, Canada) according to manufacturer’s instructions. For IL-1β and BDNF, standards and samples were tested in duplicates. For IL-1β assay, all the results below the lower limit of quantification (LLOQ) of 20 pg/mL were assigned half the LLOQ, i.e. 10 pg/mL. For the BDNF assay, all the results were above the LLOQ.

### Statistical considerations

Based on previous therapeutic intervention studies at Pharmaseed, a sample size of 15 animals per treatment group was considered to be the minimum number required to provide indicative information regarding potential drug treatment effect. In order to account for anticipated mortality in the first few days associated with transient ischemic stroke, 76 animals (~25 per group) were initially enrolled in the study and underwent surgery to induce stroke.

Except for the analysis of BDNF levels, all *P*-values reported are from statistical analysis performed by one- or two-way analysis of variance (ANOVA) for repeated measures, followed by Bonferroni post hoc test to adjust for the multiple comparisons. The Bonferroni correction was for the six comparisons that were conducted for each parameter: vehicle vs. each of two drug groups (1.5 mg/kg, 4.5 mg/kg), at each of 3 time points (Day 2, Week 4 and Week 6). One-way ANOVA was also used to compare the mNSS values at two different time points (e.g. Week 4 versus Day 2, or Week 6 versus Week 4) within an individual test group. Detailed output of the ANOVA results is provided in the S1 Appendix in the Supporting information.

Because the BDNF protein level data were not normally distributed, the BDNF statistical analyses were conducted utilizing nonparametric approaches. Specifically, the distribution-free trend test, the Jonckheere-Terpstra test, was utilized to assess for treatment-dependent dose effect across the vehicle, 1.5 mg/kg neflamapimod, and 4.5 mg/kg neflamapimod dose groups, since the result values were not normally distributed. Additionally, the Kruskal-Wallis test with Dunn’s post hoc test for multiple comparisons was performed to compare individual neflamapimod dose groups to the vehicle group. The detailed output of the BDNF statistical analysis is provided in the S2 Appendix in the Supporting information.

Except for the Jonckheere-Terpstra test, all statistical analyses were performed utilizing Prism (GraphPad Software). As there are no nonparametric tests to test dose-response in Prism, the Jonckheere-Terpstra test was programmed and analyzed utilizing S-PLUS (Tibco Software).

## Results

### Transient ischemia in rats caused significant increase in mNSS value without high incidence of mortality during the acute phase (i.e. 0–48 hours) after stroke

Seventy-six (76) young adult, 3-month old male Sprague-Dawley rats were included in the study and underwent tMCAO. In order to limit the number of animals undergoing surgery and on-study neurologic examinations on any given day, the study was conducted in four cycles of nineteen animals each that underwent tMCAO on the same day. Nine animals were lost during Day 1 and four additional animals were lost during Day 2 after the procedure, resulting in ~17% mortality during the first 48 hours post tMCAO attributable to the severity of these rat's stroke.

To confirm induction of stroke on Day 2 of each cycle, at 24 hours after reperfusion, surviving animals were subjected to neurological evaluation using the mNSS value (S1 Table [49]) that rates neurological functioning on a scale from 0 (healthy) to 18 (maximum impairment) [50]. The measured mNSS value in individual rats rose from 0 before the Day 1 tMCAO procedure to a mean±standard deviation (SD) value of 14.0±1.4 on Day 2, with individual animal mNSS values ranging from 12 to 17.

On Day 3 of each cycle, at 48 hours post stroke, the 63 surviving animals were allocated based on Day 2 mNSS values and assigned to one of three treatment groups: twice daily 1.5 mg/kg neflamapimod (22 rats initiated treatment), twice daily 4.5 mg/kg neflamapimod (21 rats initiated treatment), or twice daily vehicle (20 control rats initiated treatment) during 5.5 days every week for a total of six weeks.

The first administration of neflamapimod treatment or vehicle control administration occurred on Day 3 at 48 hours post reperfusion. Three additional rats, including one animal from the 1.5 mg/kg neflamapimod dose group and two vehicle control rats, did not survive the first two weeks after initiation of dosing. Since these additional deaths were inversely related to the dose of neflamapimod, they were attributed to the severity of their stroke. The 60 remaining rats (n = 21 in the 1.5 mg/kg and n = 21 in the 4.5 mg/kg neflamapimod groups, respectively, and n = 18 in the vehicle group) completed the planned six weeks of dosing and were included in the neurologic and behavioral evaluations at Week 4 and Week 6.

### Observations of animal health preservation including body weight gain after initiation of neflamapimod or vehicle treatment during the subacute phase after stroke

The Day 1 mean±SD body weight data for the animals assigned to each group revealed were well balanced (328.5±11.3 g for the 1.5 mg/kg and 325.3±11.1 g for the 4.5 mg/kg neflamapimod treatment group, respectively, and 328.2±9.6 g for the vehicle control group). Weekly body weight monitoring and two-way ANOVA statistics followed by Bonferroni post hoc comparisons revealed no statistically significant differences in body weight for the three study groups at any time throughout the study. The mean±SD body weight for the 3-month old rats was 327.0±10.9 g on Day 1 and all surviving animals had a mean±SD body weight 418.3±27.6 g at Week 6, and a similar body weight gain of ~27% was observed in all three test groups throughout the study period (S2 Table). These observations together with favorable results for all the daily health assessments point out that the neflamapimod and vehicle treatments were generally well-tolerated, providing no treatment-related adverse clinical signs. Moreover, these findings exclude the possibility that a difference in the general health of the rats contributed to neflamapimod treatment-mediated effects on functional recovery when compared to the effects observed in the vehicle-treated group.

### Dose-related improvement in neurologic and behavioral mNSS after initiation of neflamapimod versus vehicle treatment during the subacute phase post stroke

**Fig 1:**
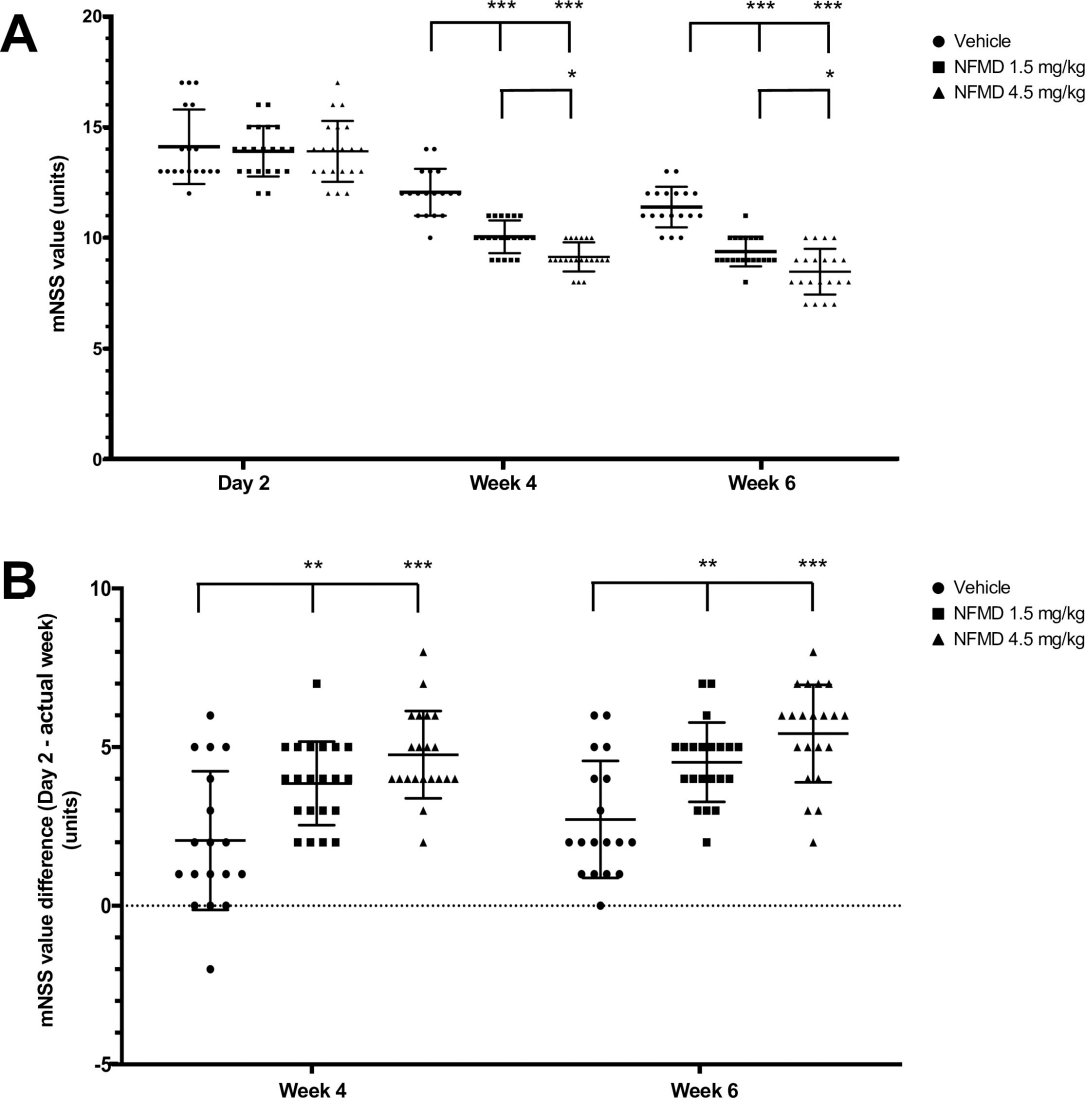
Dose-related neflamapimod (NFMD) subacute treatment effects on mNSS of rats with tMCAO. The study included three groups: vehicle (Pluronic F108; n = 18), 1.5 mg/kg neflamapimod (n = 21), and 4.5 mg/kg neflamapimod (n = 21). (A) Mean±SD mNSS value at Day 2, Week 4 and Week 6. Both at Week 4 and Week 6, the mean±SD mNSS values were decreased and significantly lower in each neflamapimod dose group when compared to vehicle. In addition, a dose response effect on improvement in neurologic function was indicated by the lower mean±SD mNSS value in the 4.5 mg/kg neflamapimod dose group when compared to that of the 1.5 mg/kg dose group. (B) Absolute changes in mNSS±SD from Day 2 to Week 4 and Day 2 to Week 6. Significant mNSS differences were observed at both time point comparisons for 1.5 or 4.5 mg/kg neflamapimod dose groups compared to vehicle compared. Significance of between group differences was assessed using a two-way analysis of variance (ANOVA) with a Bonferroni correction (*P<0.05; **P<0.01; ***P<0.001; see text for exact *P*-values>0.001).

On Day 2, the mean±SD values for mNSS were similar across the groups of stroked rats, indicating that the three randomized experimental groups were balanced a day prior to treatment initiation (Fig 1A). The measured mNSS mean±SD values on Day 2 were 13.9±1.1 in the 1.5 mg/kg and 13.9±1.4 in the 4.5 mg/kg neflamapimod dose group, and 14.3±1.7 in the vehicle control group. In light of the maximum possible mNSS value of 18 (S1 Table), all animals exhibited severe neurologic deficits prior to neflamapimod treatment initiation.

Importantly, clear differences were demonstrated between the groups treated with neflamapimod compared to the vehicle-treated group. As shown in Fig 1A, the mean±SD mNSS values were statistically significantly lower (in the 1.5 mg/kg dose group (10.0±0.7 at Week 4 and 9.4±0.7 at Week 6; ANOVA, Bonferonni post hoc test vs. vehicle at Week 4 t(37) = 6.049, *P*<0.001, and at week 6 t(37) = 5.761, *P*<0.001) and in the 4.5 mg/kg dose group (9.1±0.7 at Week 4 and 8.5±1.0 at Week 6; ANOVA, Bonferonni post hoc test vs. vehicle at Week 4 t(37) = 8.641, *P*<0.001, and at week 6 t(37) = 8.353, *P*<0.001) at both time points when compared to vehicle (see above, 12.1±1.1 at Week 4 and 11.4±0.9 at Week 6). These results document a positive effect of neflamapimod on neurologic repair following induction of ischemic stroke. Furthermore, a dose-related effect on improvement in neurologic function was indicated by the statistically significant lower mean mNSS in the 4.5 mg/kg dose group when compared to the 1.5 mg/kg dose group (ANOVA, Bonferonni post hoc test, t(40) = 2.698, *P* = 0.03 at Week 4; t(40) = 2.698, *P* = 0.03 at Week 6). Findings were similar when absolute changes in mNSS from Day 2 to Week 4 and Day 2 to Week 6 in the 1.5 mg/kg dose group (3.9±1.3 and 4.5±1.2, respectively) and the 4.5 mg/kg dose group (4.8±1.4 and 5.4±1.5, respectively) were compared to the absolute changes in the vehicle group (2.1±2.2 and 2.7±1.8, respectively), as shown in Fig 1B. Statistically significant differences in absolute change in mNSS were observed at both time point comparisons for the 1.5 mg/kg dose group (ANOVA, Bonferonni post hoc test t(37) = 3.511, *P* = 0.003 at Week 4; t(37) = 3.511, *P* = 0.003 at Week 6) and the 4.5 mg/kg dose group (ANOVA, Bonferonni post hoc test t(37) = 5.072, *P*<0.001 at Week 4; t(37) = 5.267, *P*<0.001 at Week 6) compared to the vehicle control group.

The vehicle group results for the mean±SD mNSS values (12.1±1.1 at Week 4 and 11.4±0.9 at Week 6 versus 14.3±1.7 on Day 2) imply a limited degree of spontaneous recovery of neurologic functions in these study animals from Day 2 to Week 4, as well as from Week 4 to Week 6.

### Neflamapimod-mediated improvement in motor and sensory function behavioral tests during the subacute stroke phase

**Fig 2:**
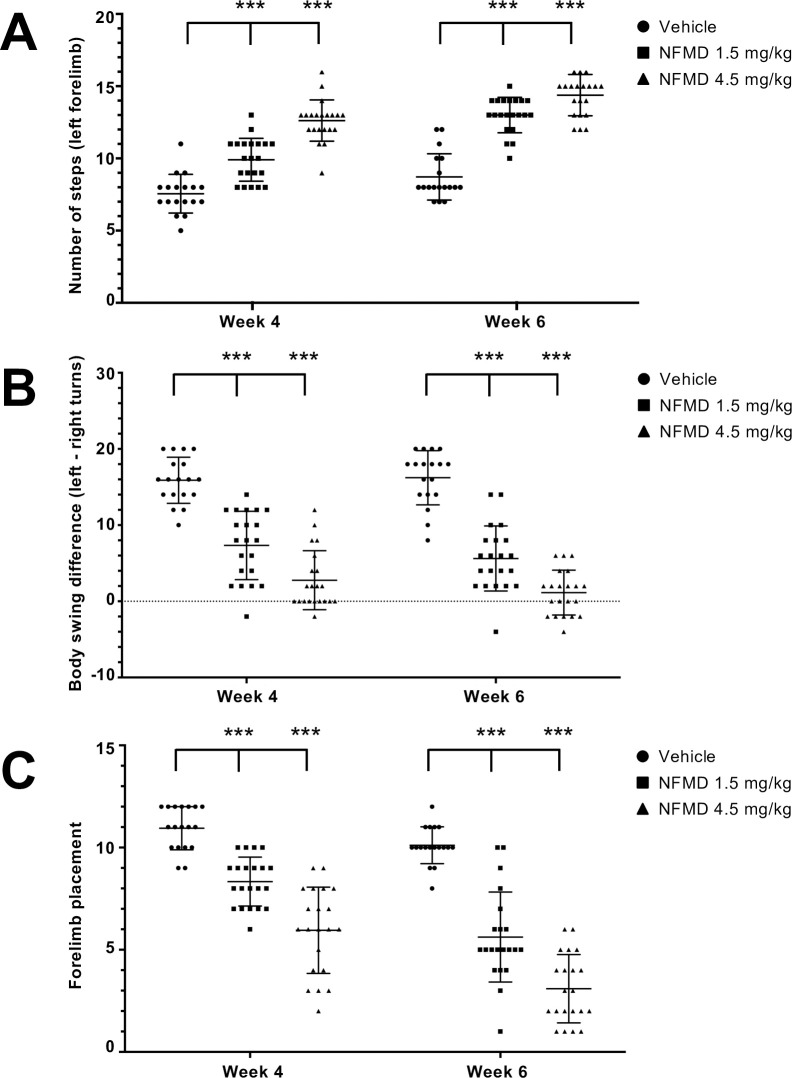
Effects of four- and six-week neflamapimod (NFMD) subacute treatment on stepping, body swing and forelimb placement tests of rats with tMCAO. Treatment groups include vehicle (n = 18), 1.5 mg/kg neflamapimod (n = 21), and 4.5 mg/kg neflamapimod (n = 21). (A) Stepping test. Rats in both neflamapimod dose groups took a significant greater mean±SD number of left forelimb steps at Week 4 and Week 6 compared to vehicle-treated animals. (B) Body swing test. Mean±SD adjusted body swing score values at Week 4 and Week 6 in the 1.5 mg/kg, 4.5 mg/kg dose groups were significantly lower when compared to vehicle group. (C) Forelimb placement test. Mean±SD forelimb placement scores at Week 4 and Week 6 in the 1.5 mg/kg and 4.5 mg/kg dose groups were significantly lower when compared to vehicle group. Significance of between group differences was assessed using ANOVA with a Bonferroni correction (*P<0.05; **P<0.01; ***P<0.001; see text for exact *P*-values>0.001).

The findings from additional behavioral evaluations of motor and sensory functions (stepping test, body swing and forelimb placement) that were performed to complement the mNSS are presented in Fig 2.

In the left forelimb stepping test, rats in both neflamapimod dose groups had a statistically significant greater mean±SD number of steps at Week 4 (9.9±1.5 in 1.5 mg/kg group and 12.6±1.4 in 4.5 mg/kg group; ANOVA, Bonferonni post hoc test vs. vehicle, t(37) = 5.103, *P*<0.001 for 1.5 mg/kg; t(37) = 9.541, *P*<0.001 for 4.5 mg/kg) and Week 6 (13.0±1.2 in 1.5 mg/kg group and 14.4±1.4 in 4.5 mg/kg group; ANOVA, Bonferonni post hoc test vs. vehicle, t(37) = 11.09, *P*<0.001 for 1.5 mg/kg; t(37) = 12.65, *P*<0.001 for 4.5 mg/kg) compared to vehicle-treated animals (7.6±1.3 at Week 4 and 8.7±1.6 at Week 6) as shown in Fig 2A. Supporting that the effects of neflamapimod were specific to neurologic recovery following tMCAO, the mean±SD number of steps for the right forelimbs were similar in all groups at Week 4 (19.1±0.4 for 1.5 mg/kg, 19.1±0.4 for 4.5 mg/kg and 18.9.7±0.2 for vehicle group) and Week 6 (19.1±0.4 for 1.5 mg/kg, 19.2±0.4 for 4.5 mg/kg and 19.1±0.2 for vehicle group), respectively, and comparable to pre-stroke baseline left forelimb (19.8±0.6 for 1.5 mg/kg, 18.8±3.3 for 4.5 mg/kg and 19.7±0.8 for vehicle group) or baseline right forelimb (20.0±0.7 for 1.5 mg/kg, 20.1±0.7 for 4.5 mg/kg and 19.7±0.8 for vehicle group) values.

Additional results from the body swing (BSW) tests indicate a more prominent motor recovery in neflamapimod-treated animals. The mean±SD adjusted BSW score in the tests at Week 4 and Week 6 in the 1.5 mg/kg (6.8±4.6 and 5.6±4.2, respectively; ANOVA, Bonferonni post hoc test vs. vehicle, t(37) = 7.511, *P*<0.001 at Week 4; t(37) = 8.75, *P*<0.001 at Week 6) and 4.5 mg/kg (2.8±3.9 and 1.1±2.9, respectively; ANOVA, Bonferonni post hoc test vs. vehicle, t(37) = 10.9, *P*<0.001 at Week 4; t(37) = 12.46, *P*<0.001 at Week 6) dose groups were all statistically significantly lower when compared to vehicle group (15.9±3.0 and 16.2±3.6, respectively) at the same time points, as presented in Fig 2B.

Finally, the mean±SD forelimb placement scores in the forelimb placement tests at Week 4 and Week 6 in the 1.5 mg/kg (8.3±1.2 and 5.5±2.2, respectively; ANOVA, Bonferonni post hoc test vs. vehicle, t(37) = 4.941, *P*<0.001 at Week 4; t(37) = 8.742, *P*<0.001 at Week 6) and 4.5 mg/kg (6.0±2.1 and 3.1±1.7, respectively; ANOVA, Bonferonni post hoc test vs. vehicle, t(37) = 9.312, *P*<0.001 at Week 4; t(37) = 13.3; *P*<0.001 at Week 6) neflamapimod dose groups were also statistically significantly lower when compared to vehicle group (10.9±1.1 and 10.1±0.9, respectively), as shown in Fig 2C. The forelimb placement test scored the above-mentioned mean±SD forelimb placement scores positive values for the affected left forelimbs post stroke only. All the forelimb placement test scores for the left and right forelimbs at baseline, as well as all the values for the right forelimbs at Week 4 and Week 6 were zero. The lower forelimb placement scores observed in the neflamapimod-treated animals suggest a treatment effect of neflamapimod on recovery of somatosensory function following ischemic stroke.

### Study termination biomarker evaluation showing no significant effect of neflamapimod treatment on IL-1β production, while providing evidence for dose-dependent neflamapimod treatment-associated increase in brain BDNF protein levels

A total of 18 tissue samples for the right and the left brain hemisphere from each animal per group, plus the standard curve, were the maximum number of assays that could be included on the ELISA plate that was utilized to evaluate the effects of neflamapimod on IL-1β and BDNF biomarkers after study termination. Importantly, all brain tissue samples from the vehicle group were included in this analysis. However, since both the 1.5 mg/kg and the 4.5 mg/kg neflamapimod dose group consisted of 21 animals, the right and left brain tissue samples from three animals each had to be omitted. Selection of the samples to be omitted from the two neflamapimod dose groups was made arbitrarily by the technician in the ELISA lab who was blinded to other data. It was determined in advance, that the right and left brain tissue samples from the 17th, 18th, and 19th animal (by animal number, lowest to highest) in each of the two neflamapimod dose groups would not be analyzed.

The ELISA signal in the IL-1β assay in the unaffected left brain hemisphere were below the level for noise in the assay (i.e. below LLOQ of 20 pg/mL) in all rats in all groups. However, 9 of 18 animals in the each of the vehicle and 1.5 mg/kg neflamapimod groups and 6 of 18 animals in the 4.5 mg/kg neflamapimod group had quantifiable IL-1β levels above 20 pg/mL in the injured right brain hemisphere, indicating that despite being six weeks from acute the stroke there was still detectable residual inflammation in a substantial percentage of the animals. Quantifiable IL-1β levels ranged from 21.3 pg/mL to 203.5 pg/mL, though all but three rats had levels below 100 pg/mL (S3 Table). The mean±SD IL-1β levels in the right hemisphere was 44.3.3±55.7 pg/ml in the vehicle group, 30.8±25.9 pg/ml in the 1.5 mg/kg neflamapimod group and 28.5±32.4 pg/ml in the 4.5 mg/kg group; with no statistically significant difference between these groups.

**Fig 3:**
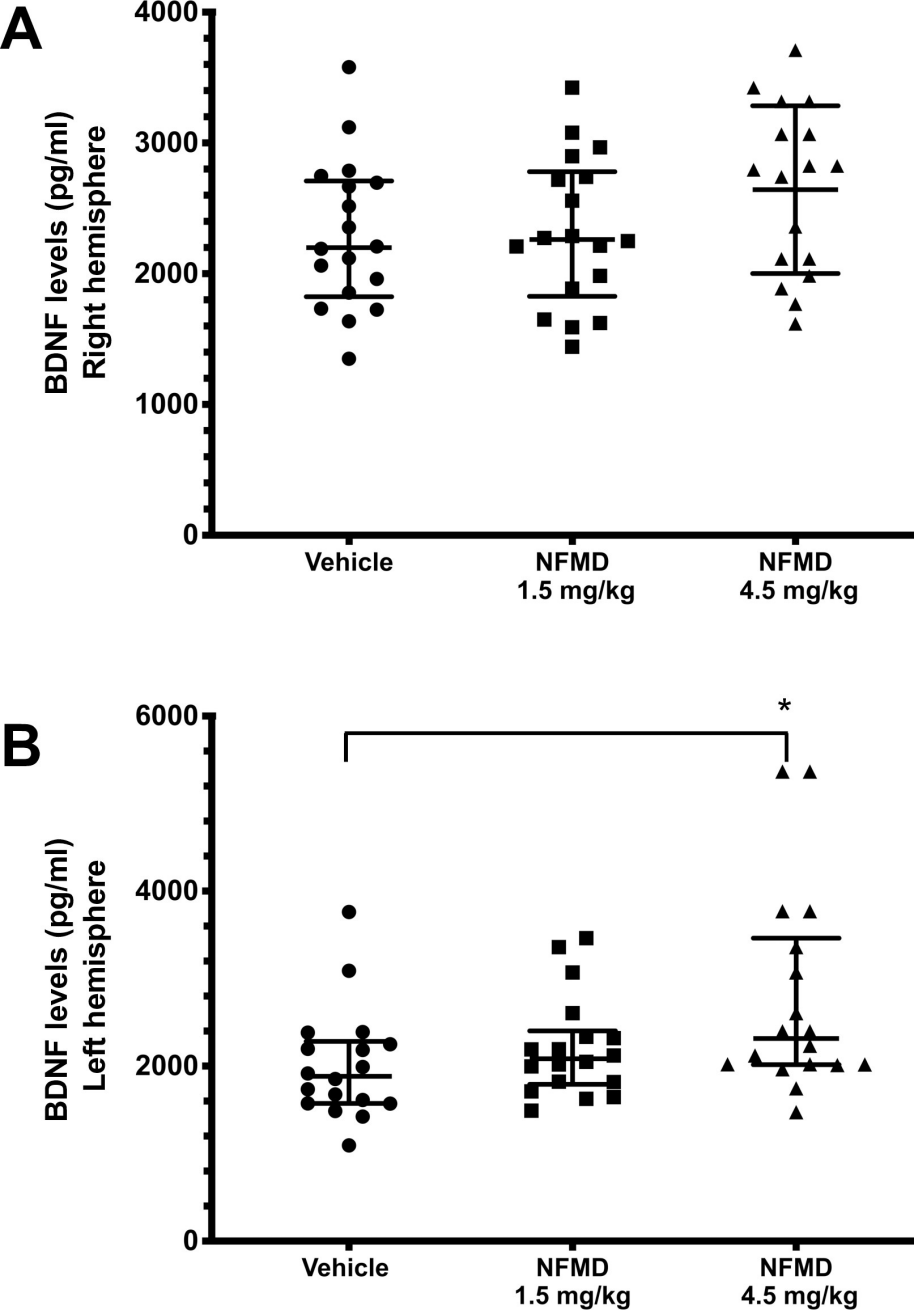
Dose-related neflamapimod (NFMD) subacute six-week treatment effects on rat brain BDNF levels analyzed on Day 44 post tMCAO. Brain homogenate samples from 18 animals in each treatment group (vehicle, 1.5 mg/kg neflamapimod, and 4.5 mg/kg neflamapimod) were prepared two days after the six-week treatment period and analyzed by BDNF ELISA. In the analysis of the right brain hemisphere within the 4.5 mg/kg neflamapimod group, there was an accidental assay failure for one sample, so BDNF ELISA results exist for 17 animals only. The median BDNF value and the 25^th^ and 75^th^ percentile are presented for each different group. (A) Right hemisphere (tMCAO): A significant neflamapimod dose trend by the Jonckheere-Terpstra test for higher BDNF levels was observed (*P*<0.05) for across the three groups. (B) Left hemisphere (non-injured side): The Jonckheere-Terpstra test indicated a significant neflamapimod dose trend for higher BDNF levels in the left hemisphere (*P*<0.01). BDNF levels were also significantly higher in the 4.5 mg/kg neflamapimod group when compared with the vehicle control group (*P*<0.05). See text for exact *P*-values >0.001.

BDNF protein was detectable in both brain hemispheres of all animals on Day 44 post stroke as shown in Fig 3, which presents the median and the lower (25^th^ percentile) and upper (75^th^ percentile) interquartile range (IQR) values for the different groups. Within each brain hemisphere there was a significant dose-related effect of neflamapimod for increasing BDNF protein levels (Jonckheere-Terpstra test: J = 2.595, *P* = 0.0047 and J = 1.673, *P* = 0.047 for the left (Fig 3B) and right (Fig 3A) hemisphere, respectively). In addition, in the left hemisphere (non-affected side), BDNF levels were statistically significantly higher in the 4.5 mg/kg group (median 2315 pg/ml (IQR: 2016–3463 pg/ml)) than in the vehicle group (median 1884 pg/ml (IQR: 1573–2283 pg/ml)) based on the results of the Kruskal-Wallis test with Dunn’s post hoc test for multiple comparisons (Z = 2.569; *P* = 0.0204). This is visualized by the horizontal line on top with the two drop downs at its ends, and the star above the 4.5 mg/kg neflamapimod dose group in Fig 3B. The left hemisphere BDNF levels in the 1.5 mg/kg group (median 2085 pg/ml (IQR: 1792–2402 pg/ml)) were intermediate to those in the vehicle and the 4.5 mg/kg group, although not statistically significantly different from the vehicle group when the 1.5 mg/kg and vehicle groups were compared directly.

## Discussion

The results for the daily general health assessments, the weekly body weight measurements, the neurologic recovery assessments on Day 2, at Week 4 and at Week 6 post stroke, and the terminal biomarker measurements on Day 44 in this rat tMCAO study taken together led to the following seven key observations for evaluation of delayed and prolonged neflamapimod treatment. (1) Neflamapimod treatment dose-dependently and continually improved neurologic and behavioral outcomes assessed by mNSS and specific measures of sensory and motor function post stroke (Figs 1 and 2). (2) Neflamapimod dose-dependently increased terminal brain BDNF protein as a neurogenic factor biomarker measure for beneficial effects on synaptic plasticity (Fig 3). (3) Neflamapimod and vehicle treatments were generally well-tolerated throughout the study, providing no treatment-related adverse clinical signs. (4) There was an average body weight gain of 27% in all treatment groups throughout the six-week study period (S2 Table) and there was no difference observed in the general health of the rats that would have contributed to any neflamapimod versus vehicle treatment-mediated functional recovery. (5) Slight, albeit statistically significant spontaneous recovery over time was also observed in the vehicle control group (an average ~19% decrease in mNSS value within six weeks; Fig 1) implying that neflamapimod *in vivo* efficacy may enhance a biologic process, like neural or synaptic plasticity, that is already active. (6) Generally, the functional recovery was slow and steady in all neflamapimod- or vehicle-treated animals throughout the prolonged study period (Figs 1 and 2). (7) There were signs for unresolved IL-1β-mediated chronic inflammation in the injured brain hemisphere of a subset of animals, however, no neflamapimod effect on IL-1β production was apparent at study termination (S3 Table). Taken together, the results for this research study provide evidence that there is an additional clinical opportunity for p38α inhibitors in ischemic stroke since neflamapimod treatment initiated outside the neuroprotection time window in a rat tMCAO model promoted recovery.

The most important finding of this study is that the delayed and prolonged treatment with neflamapimod resulted in dose-related neurologic and behavioral improvements versus vehicle control, when this p38α inhibitor was administered orally to rats with tMCAO in an experimental therapeutic paradigm to enhance synaptic plasticity and functional recovery during the recovery phase after ischemic stroke. To this end, neflamapimod treatment was started at 48 hours after reperfusion at a time when the acute stroke phase was considered complete, since this represents a time point that is outside the previously characterized neuroprotection window of less than 24-hours post tMCAO-induced stroke for a p38α inhibitor [30]. In the most relevant previously published study, the p38 MAPK inhibitor SB203580 was administered via intracerebroventricular injection 30 minutes pre-tMCAO, or at 6, 12 or 24 hours post tMCAO to Sprague Dawley rats and demonstrated a decrease in infarct volume that was associated with anti-inflammatory effects in the injured brain and improved recovery from neurologic deficit measured on Day 2 post stroke for all the treatment initiations up to 12 hours post tMCAO, whereas no beneficial effect was observed for the treatment initiation at 24 hours post tMCAO [30]. The observation that a 12-hour time window for the start of p38α inhibition, but not a 24-hour window allowed for neuroprotective effects in the rat tMCAO-induced stroke model supports the selection of 48-hours as the time point for initiation of subacute phase neflamapimod dosing in the current study, since the results by Piao *et al*. [30] indicated that it is too late a time point for a p38α inhibitor in this type of model to lead to reduction of neuronal loss as a mechanism for impacting functional outcome.

While infarct size was not measured in this study (the harvested brain was instead utilized to measure protein biomarkers), the acute mortality rate, the high mNSS score at Day 2, and modest spontaneous recovery in the vehicle animals are observations that are consistent with prior validation studies of this model conducted at Pharmaseed, in which the infarct size ranged from 27 to 48% of the injured hemisphere, and reduction in blood flow was between 16 and 31%. Mortality of up to 25% within 24 hours after stroke onset in experimental stroke models is quite common [51–53] and mortality has been reported to increase up to 33% in the following days due to edema [54, 55]. In this tMCAO study, 13 out of 76 animals (~17%) were lost within the 48 hours post stroke, and 3 more during the first two weeks of treatment, resulting in ~21% mortality within the first 16 days after tMCAO. These results are well within the range of the expected mortality results for rodent stroke models in which moderate to large stroke is induced [51–55]. The level of improvement in mNSS scores in the vehicle group (19%) is lower than the range of 30–50% improvement seen in reported studies in control groups over four to six weeks after tMACO in rats [56–60]. However, the mean mNSS scores at 24 hours after stroke inductions were between 9 and 11 (several excluded animals with mNSS scores of >12) [56–60] compared to a mean mNSS of >14 in the current study, again supporting that the animals in the current study had greater infarct volume and were sicker going into the recovery phase than those in the other studies.

The current study is clearly different from all previously reported studies of other small molecule p38 MAPK inhibitors in various experimental stroke models [30, 33–37] both in terms of the delay in treatment initiation to 48 hours post reperfusion and the six-week prolonged dosing duration and overall treatment study focus. All previously reported studies of p38 MAPK inhibitors that most potently inhibit the p38α isoform, including SB203580 [30], SB239063 [33, 34], RWJ67657 [36], or VCP979 [37], involved compound administration before or soon after the experimental stroke during the acute phase, with treatment initiations at different time points, ranging from as early as 1 hour pre stroke to as late as 24 hours post stroke. Additionally, all prior p38α inhibitor studies had a focus on demonstrating neuroprotective effects that resulted in significant reduction of infarct volume (assessed as early as 6 hours post stroke) and subsequent improvements of neurological defects assessed at 24 hours [33, 34], 2 days [30], 7 days [37] or 14 days post stroke [36]. The two most recent p38α inhibitor studies [36, 37] were also the two longest ones prior to the present study. Interestingly, they did demonstrate the validity of mNSS [50] as a readout for functional recovery attributable to neuroprotection via p38α inhibition, that was measurable during the subacute phase after photothrombotic ischemic stroke in mice at one and two weeks, respectively [36, 37]. The results of this neflamapimod study extend these positive mNSS effect observations for a p38α inhibitor to a rat tMCAO model. Evaluation of mNSS [50] and additional functional tests (stepping test, body swing test, forelimb placement test) to assess the sensory motor deficit after stroke together with BDNF protein biomarker effects were deemed sufficient to evaluate and demonstrate the beneficial *in vivo* efficacy of neflamapimod.

Since the present study represents a first both for initiating the dosing of a potent and selective p38α inhibitor in the subacute phase after rodent tMCAO-induced stroke (i.e. outside the time window for neuroprotection), as well as for dosing this type of a small molecule for a prolonged time period of six weeks, it is a challenge to find directly supportive subacute or chronic stroke phase studies. It is reassuring that the functional recovery demonstrated in this study is consistent with a report by Umezawa *et al*. [61] who demonstrated that selective inhibition of p38α by SB239063 improved locomotor recovery after SCI in mice. Although in a different disease context, this study may be most relevant since Umezawa *et al*. [61] also utilized a genetic approach (i.e. a heterozygous knockout of MAPK14) to identify the p38α specificity of the effect. Generally, small molecule treatments in the rodent cerebral ischemia model start before the 48-hour time point after reperfusion, as already discussed for all the previously reported p38α inhibitor studies [30, 33–37]. Those studies are not inconsistent with this neflamapimod study, only different in that the neurologic recovery effects therein are clearly associated with a primary acute phase neuroprotective effect of the p38α inhibitors [30, 33–37]. A review of the experimental stroke literature, however, revealed that the results of all these studies are in conflict with several reports that have associated elevated p38 MAPK activity, including p38α activity, with a neuroprotective role during and after stroke, either in the acute or subacute phase [62–68]. A major caveat is that several of these differing studies used the small molecule SB203580 at a relatively high concentration and did not address the possibility of blocking p38 MAPK isoforms other than p38α, such as p38β, or other kinases (e.g. GAK, RIPK2, NLK, JNK3, CSNK1D and others) with this less potent and much less selective p38α inhibitor tool compound [69]. As an example, SB203850 through inhibiting casein kinase Iδ and ε blocks WNT-stimulated β-catenin signaling [70, 71], which might account for compound effects on neurogenesis, therefore, render claims for p38 attributed activities invalid [65]. The current results with a selective p38α inhibitor help to resolve this conflict and indicated that activation of p38α in the recovery phase is deleterious.

Oral neflamapimod was dosed at 1.5 and 4.5 mg/kg administered in 1% Pluronic F108 vehicle twice daily on 5.5 days per week for six weeks in the present rat tMCAO study. These two neflamapimod dose levels in this vehicle were selected because their twice daily administration for three weeks was previously demonstrated to be pharmacologically active in aged rats with cognitive deficits attributed to chronic inflammation-induced, IL-1β-mediated impairment of synaptic plasticity [45]. The results obtained in this tMCAO study corroborate that these two neflamapimod dose levels are pharmacologically active in rats. Unlike the aged rat study, in which the 4.5 mg/kg dose did not lead to better cognitive results compared to the 1.5 mg/kg dose [45], the beneficial effects by neflamapimod resulting in neurologic and behavioral improvements after stroke increased in a dose-related manner and enhanced the slight spontaneous recovery observed in the vehicle control group.

The IL-1β results of the present rat tMCAO study, though limited to one time point, are to our knowledge the first time that such results have been reported this late out after the acute stroke event in this model. The results showed residual elevation of IL-1β on the stroked side of the brain, but not on the unaffected side. IL-1β in the injured brain hemisphere was measurable in 50% of animals in the vehicle- and low-dose neflamapimod-treated group as well as in 30% of the animals in the high-dose neflamapimod-treated group at the end of the study. This together with the observed beneficial neurologic effects by neflamapimod suggests for the first time that chronic inflammation may play a deleterious role in the recovery process in the rat tMCAO stroke model. This is a finding that supplements previously reported IL-1β results during the acute phase in this type of model [13, 72, 73]. Although this single time point analysis appears to exclude a neflamapimod effect on IL-1β production, it does not preclude a neflamapimod effect on IL-1β signaling (see further discussion below).

Terminal measurements of brain BDNF protein demonstrated that six-week neflamapimod treatment resulted in dose-dependent increases of this neurotrophic factor in both the injured and uninjured hemisphere. BDNF is a key regulator of plasticity both in the healthy and injured brain which has been recognized as a key regulator of rehabilitation- and activity-induced functional and motor recovery, respectively, after stroke [74–76]. BDNF is reported to have a critical role in promoting recovery after stroke as a crucial signaling molecule that mediates adaptive brain plasticity [22, 77–80]. Increased BDNF levels in perilesional areas have been observed with interventions that improve functional recovery post stroke [81–83]. Conversely, attenuation of brain BDNF levels or effects following cerebral ischemia results in reduced neuroplastic changes or decreased recovery of function, both in spontaneous and in rehabilitation-induced recovery scenarios [79, 84, 85]. Since regenerative roles have been attributed to BDNF in preclinical models of stroke [79, 86–89], upregulation of BDNF may be a plausible contributor to the neflamapimod-induced functional recovery observed after the ischemic stroke.

How BDNF elevation is linked to p38α inhibition by neflamapimod in this experimental stroke model will need to be determined in a follow-up mechanistic study. Our favored hypothesis is that the BDNF levels might be interpreted as a marker of a more general effect on IL-1β signaling [19] that could result from p38α inhibition [18, 21]. In such a hypothetical mechanistic model, the observation of inhibition of IL-1β signaling without impacting IL-1β production would be consistent with previously observed aged rat results demonstrating that neflamapimod is more potent at inhibiting IL-1β signaling than at inhibiting IL-1β production [45]. While this stroke study was not intended to address the exact mechanism of action of neflamapimod, the observation that BDNF protein was increased in both brain hemispheres at study termination is nevertheless supporting the underlying hypothesis that functional recovery was associated with enhancing synaptic plasticity.

Taking into account the design of the study that precludes an effect through neuroprotection, the absence of an anti-inflammatory effect, and the possibility of an effect on IL-1β signaling, combined with the scientific literature regarding p38 MAPK-mediated deleterious effects of IL-1β on synaptic plasticity [21, 25], the results herein imply a model in which IL-1β would limit functional recovery after stroke via p38α-mediated impairment of neural and synaptic plasticity. The results in this neflamapimod-mediated stroke recovery study are also consistent with studies in other disease contexts in which activation of p38 MAPK, particularly the alpha isoform, is associated with impaired synaptic plasticity [90, 91]. Further, the non-clinical to clinical translational potential with neflamapimod as a p38α inhibitor is that, similar to the observed slight spontaneous recovery observed in the vehicle control group, neural and synaptic plasticity has been argued to be active in humans and at least partially effective in recovery after stroke [92]. Enhancing a biologic process like neural or synaptic plasticity, that is already active, rather than targeting a process that may not represent an intrinsic recovery pathway, such as neurogenesis, should at least theoretically be more likely to have a clinical effect. For this reason, the opportunity of translational success with p38α inhibition to promote recovery following stroke by enhancing plasticity would be expected to be higher than for neuroprotection.

Potentially the most important consideration for clinical translation when targeting recovery from stroke is the practical consideration of time window for therapeutic intervention. The narrow time window after onset of ischemia that is required of neuroprotective approaches has posed an insurmountable challenge for clinical development [93]. Stroke patients do not often present within the required first few hours and, even within the initial 24 hours post stroke, the clinical presentation does not allow one to assess the true size and severity of the stroke. Therefore, any clinical study that starts treatment of patients within the first day of the stroke has a highly variable and heterogeneous patient population and thus requires a large sample size to demonstrate clinical effects. These restrictions preclude the ability to demonstrate clinical proof-of-concept in phase 2 clinical testing. In contrast, a time window of 24 to 48 hours after stroke allows patients’ clinical course to have stabilized and allows time for a clinical exam and a diffusion-MRI scan to precisely determine the location and extent of the stroke before starting treatment. In addition, a reasonable prognostic indication of the extent of recovery that a patient will attain with or without intervention can be assessed. As a result, starting treatment in a clinical study at 48 hours or later after stroke allows for the inclusion of more homogenous sub-populations of patients and increases statistical power with fewer subjects; therefore, definitive clinical-proof-of-concept could potentially be demonstrated within a phase 2 clinical study.

As immunosuppression with T lymphocyte depletion in the systemic circulation is an intrinsic component of the clinical syndrome during the subacute phase after ischemic stroke [94], a potential concern of targeting IL-1β and the innate immune system is an increased risk of infection due to further suppressing the immune system systemically. In the case of targeting IL-1β with selective inhibition of p38α, this particular concern is low because in transgenic models p38α is dispensable for T-cell development and activation [95, 96]. Indeed, for T cell development the p38 MAPK isoforms primarily involved in T lymphocyte development are the minor isoforms, p38γ and p38δ [97]. Consistent with this notion, in a 3503-patient twelve-week treatment phase 3 trial of the p38α/β inhibitor losapimod in patients with acute myocardial infection the incidence of infection was 2.7% and 2.4%, and of opportunistic infection 0.3% and 0.4%, in losapimod and placebo treatment groups, respectively [98]. The risk of systemic immunosuppression is expected to be further attenuated with neflamapimod as it preferentially distributes to the CNS, with plasma drug levels in peripheral blood obtained after dosing in humans that have CNS activity being three- to five-fold lower than those required to for pharmacologic activity [42]. Further, neflamapimod has been administered to more than 300 patients and volunteers at doses up to ten-fold-higher than anticipated doses in the stroke setting, and up to six months, and to date there has been no evidence of an increase in infections (EIP Pharma Inc., data on file).

After our studies were completed, additional preclinical and clinical study results have been reported on the potential of therapeutically targeting IL-1β [99]. In particular, a single-center 80-patient phase 2 placebo-controlled clinical study has been reported on the effects of subcutaneous interleukin-1 receptor antagonist (IL-1Ral anakinra) which blocks both IL-1α and IL-1β signaling [100]. IL-1RA was administered to patients starting within 5 hours of an acute stroke, every 12 hours for six doses. The study met its primary endpoint by reducing serum concentrations of interleukin-6, as well of c-reactive protein levels (i.e. reduced inflammatory markers). However, there was a non-significant trend towards worse functional outcomes at 90 days, as measured by the modified Rankin scale. The authors attribute the potential deleterious effect on functional recovery on a negative interaction with alteplase, as in a previous phase 2 study of IL-1RA conducted prior to widespread use of thrombolysis they had seen a positive trend on outcomes at 90 days. An additional consideration is that IL1-Ra inhibits all signaling, whether beneficial or deleterious, of both IL-1α and IL-1β; while a later report demonstrated that delayed administration of IL-1α to mice after stroke induced by filament-based MCAO ameliorated functional deficit and promoted neural repair [101]. In contrast, as discussed in the introduction, inhibition of p38α would be expected to only block the deleterious effects of IL-1 on synaptic plasticity that are mediated by the ubiquitous form of IL1-RAP, while preserving signaling through the neuron-specific form of IL1-RAP that enhances synaptic plasticity [23–25].

There are some limitations to this research stroke study, since only one treatment duration was chosen, and functional outcomes were measured at the Week 4 and Week 6 timepoints after the stroke. It cannot be predicted whether longer neflamapimod treatment duration with additional analyses would have led to further mNSS improvement and continued recovery during the chronic phase, and the exact timing of the onset of neflamapimod action on functional recovery was not determined. Neflamapimod treatment was initiated at 48 hours after reperfusion, at a time point that was considered subacute post stroke in light of the prior negative experiences with other p38α inhibitors and numerous neuroprotective agents when they were administered at 24 hours or more post stroke, which implies an exceedingly low likelihood that neuroprotection plays a role in promoting functional recovery, but without accompanying histologic analyses an effect of neflamapimod on neuronal loss cannot be formally excluded. Further, while we believe that it is highly probable that the functional recovery mediated by neflamapimod was due to enhancement of plasticity mechanisms, in the absence of technologies that can measure synaptic function *in vivo* in real-time, there is no means to directly verify that assumption. Similarly, while the known activity of IL-1β to impair synaptic plasticity via p38α implies that inhibition IL-1β activity is a major contributor to the neflamapimod clinical activity, there is no means to directly confirm that link *in vivo*, or to exclude other potential mechanisms. Finally, in the current study, young (3-months old) male animals were utilized. Although neural and synaptic plasticity recovery functions appear to be active in aged animals and are also at least partially preserved in elderly patients [102], in order to improve clinical translation, a replication and extension of this study to include females, aged animal and animals with co-morbidities may be required since gender, age and reduced health condition (e.g. illnesses, diseases, disorders, health problems) may affect development of ischemic damage and resulting behavioral deficits in patients [103].

## Conclusions

Here prolonged, six-week oral administration of the p38α inhibitor neflamapimod, with treatment initiation starting at 48 hours post reperfusion that is outside the previously characterized neuroprotection window for p38α inhibitors, resulted in dose-related significant neurologic recovery and improvement of motor and sensory functions measured at four and six weeks post stroke. Additionally, dose-related increases of the neurogenic factor BDNF in the brain as a potential biomarker for neflamapimod effects on synaptic plasticity were observed at termination of the study on Day 44. Thus, neflamapimod use offers the possibility of being effective when given at a later time after a completed stroke by promoting functional recovery.

## Supporting information

S1 Table Modified Neurological Severity Score (mNSS) tests and scoring values. (PDF)

S2 Table Body weight monitoring of the three treatment groups, including vehicle control, 1.5 mg/kg neflamapimod (NFMD), and 4.5 mg/kg NFMD throughout the study. (PDF)

S3 Table IL-1β (pg/ml) levels in the injured right brain hemisphere on Day 44 post stroke. (PDF)

S1 Appendix Statistical analysis details. (PDF)

S2 Appendix BDNF statistical analysis details. (PDF)
