# Supplementary material for: Continuous administration of a p38α inhibitor during the subacute phase after transient ischemia-induced stroke in the rat promotes dose-dependent functional recovery accompanied by increase in brain BDNF protein level
Source: PLoS One. 2020 Dec 4;15(12):e0233073. doi: 10.1371/journal.pone.0233073 (PMC7717516; doi:10.1371/journal.pone.0233073)
Supplement: S1 Table — (PDF) [file pone.0233073.s001.pdf]

S1 Table. Modified Neurological Severity Score (mNSS) tests and scoring values.

**Motor test score values and descriptions (Normal score = 0; maximum possible summary score = 6)**	
0 or 1*	Flexion of forelimb after raising rat by the tail
0 or 1*	Flexion of hindlimb after raising rat by the tail
0 or 1*	Head moved >10° to vertical axis within 30 seconds after raising rat by the tail
0	Normal walk after placing rat on the floor
1	Inability to walk straight after placing rat on the floor
2	Circling toward paretic side after placing rat on the floor
3	Falls down to paretic side after placing rat on the floor
**Sensory test score values and descriptions (Normal score = 0; maximum possible summary score = 2)**	
0 or 1*	Placing test (visual and tactile test)
0 or 1*	Procioceptive test (deep sensation, pushing paw against table to stimulate limb muscles)
**Beam and balance test score values and descriptions (Normal score = 0; maximum possible summary score = 6)**	
0	Balances with steady posture
1	Grasps side of beam
2	Hugs beam and 1 limb falls down from beam
3	Hugs beam and 2 limbs fall down from beam, or spins on beam (60 seconds)
4	Attempts to balance on beam, but falls off (> 40 seconds)
5	Attempts to balance on beam, but falls off (> 20 seconds)
6	Falls off; no attempt to balance or hang on to beam (< 20 seconds)
**Reflex absence and abnormal movements test score values and descriptions (Normal score = 0; maximum possible summary score = 4)**	
0 or 1*	Pinna reflex (head shakes when auditory meatus is touched with cotton)
0 or 1*	Corneal reflex (eye blink when cornea is lightly touched with cotton)
0 or 1*	Startle reflex (motor response to a brief noise from snapping a clipboard paper)
0 or 1*	Seizure, myoclonus, myodystony

*Score value of 1 was given for the inability to perform a test, or for the lack of a tested reflex, or for abnormal movement as described by Chen *et al.* [49].
