# Supplementary material for: Continuous administration of a p38α inhibitor during the subacute phase after transient ischemia-induced stroke in the rat promotes dose-dependent functional recovery accompanied by increase in brain BDNF protein level
Source: PLoS One. 2020 Dec 4;15(12):e0233073. doi: 10.1371/journal.pone.0233073 (PMC7717516; doi:10.1371/journal.pone.0233073)
Supplement: S2 Table — (PDF) [file pone.0233073.s002.pdf]

S2 Table. Body weight monitoring of the three treatment groups, including vehicle control, 1.5 mg/kg neflamapimod (NFMD), and 4.5 mg/kg NFMD throughout the study.

Body weight monitoring time	Body weight Vehicle Mean±SD (g)	Body weight 1.5 mg/kg NFMD Mean±SD (g)	Body weight 4.5 mg/kg NFMD Mean±SD (g)
**Day 1**	328.2±9.6	328.5±11.3	325.3±11.1
**Week 1**	309.2±36.3	325.7±26.8	321.1±27.2
**Week 2**	342.2±34.2	353.9±24.9	353.3±23.8
**Week 3**	369.2±30.7	378.5±23.1	374.8±21.4
**Week 4**	388.6±30.5	397.3±25.1	393.9±25.5
**Week 5**	403.6±31.4	412.4±25.6	407.5±21.1
**Week 6**	415.0±34.5	421.5±26.8	417.8±23.0
