# Supplementary material for: Continuous administration of a p38α inhibitor during the subacute phase after transient ischemia-induced stroke in the rat promotes dose-dependent functional recovery accompanied by increase in brain BDNF protein level
Source: PLoS One. 2020 Dec 4;15(12):e0233073. doi: 10.1371/journal.pone.0233073 (PMC7717516; doi:10.1371/journal.pone.0233073)
Supplement: S3 Table — (PDF) [file pone.0233073.s003.pdf]

S3 Table. IL-1β (pg/ml) levels in the injured right brain hemisphere on Day 44 post stroke.

Vehicle		1.5 mg/kg NFMD		4.5 mg/kg NFMD	
**Rat Number**	**IL-1β (pg/ml)**	**Rat Number**	**IL-1β * (pg/ml)**	**Rat Number**	**IL-1β * (pg/ml)**
2	37.2	1	35.2	9	10
8	57.5	4	10	12	10
16	49.3	5	33.2	14	85.0
20	24.4	18	62.5	15	116.8
28	10	23	10	22	10
29	32.8	27	39.9	24	10
33	203.5	31	10	25	45.1
38	10	40	10	26	10
43	166.1	41	10	32	70.8
45	70.8	42	10	34	10
51	65.7	44	67.8	36	10
52	10	46	53.1	39	10
54	10	50	21.3	47	21.3
56	10	53	10	57	54.6
62	10	55	10	58	10
64	10	60	10	59	10
69	10	73	10	75	10
77	10	74	8.9	76	10
**Mean**	**44.3**	**Mean**	**30.8**	**Mean**	**28.5**
**SD**	**55.7**	**SD**	**25.9**	**SD**	**32.4**

Note : IL-1β values below the LLOQ value of 20 pg/ml were set to 10 pg/mL (i.e. half of LLOQ).
