# Supplementary material for: Continuous administration of a p38α inhibitor during the subacute phase after transient ischemia-induced stroke in the rat promotes dose-dependent functional recovery accompanied by increase in brain BDNF protein level
Source: PLoS One. 2020 Dec 4;15(12):e0233073. doi: 10.1371/journal.pone.0233073 (PMC7717516; doi:10.1371/journal.pone.0233073)
Supplement: S1 Appendix — (PDF) [file pone.0233073.s004.pdf]

Statistical analysis details

Table A. Body weight (BW) analysis by group during study

Two-way ANOVA				
Source of Variation	% of total variation	*P* value		
Interaction	0.30	0.9864		
Column Factor	0.72	0.0109		
BW	67.24	< 0.0001		
Source of Variation	P value summary	Significant?		
Interaction	ns	No		
Column Factor	*	Yes		
BW	***	Yes		
Source of Variation	Df	Sum-of-squares	Mean square	F
Interaction	12	2485	207.1	0.3162
Column Factor	2	5986	2993	4.569
BW	6	557841	92974	141.9
Residual	402	263304	655.0	
Number of missing values	39			
Bonferroni posttests				
Vehicle vs NFMD 1.5mg/kg				
BW	Vehicle	NFMD 1.5mg/kg	Difference	95% CI of diff.
Day 1	328.0	329.0	1.000	-22.18 to 24.18
Week 1	309.0	326.0	17.00	-5.094 to 43.09
Week 2	342.0	354.0	12.00	-12.09 to 36.09
Week 3	369.0	378.0	9.000	-15.09 to 33.09
Week 4	389.0	397.0	8.000	-16.09 to 32.09
Week 5	404.0	412.0	8.000	-16.09 to 32.09
Week 6	415.0	422.0	7.000	-17.09 to 31.09
BW	Difference	t	*P* value	Summary
Day 1	1.000	0.1265	*P*>0.05	ns
Week 1	19.00	2.311	*P*>0.05	ns
Week 2	12.00	1.460	*P*>0.05	ns
Week 3	9.000	1.095	*P*>0.05	ns
Week 4	8.000	0.9732	*P*>0.05	ns
Week 5	8.000	0.9732	*P*>0.05	ns
Week 6	7.000	0.8515	*P*>0.05	ns
Vehicle vs NFMD 4.5mg/kg				
BW	Vehicle	NFMD 4.5mg/kg	Difference	95% CI of diff.
Day1	328.0	325.0	-3.000	-26.44 to 20.44
Week 1	309.0	321.0	12.00	-10.09 to 38.09

Week 2	342.0	353.0	11.00	-13.09 to 35.09
Week 3	369.0	375.0	6.000	-18.09 to 30.09
Week 4	389.0	394.0	5.000	-19.09 to 29.09
Week 5	404.0	408.0	4.000	-20.09 to 28.09
Week 6	415.0	418.0	3.000	-21.09 to 27.09
BW	Difference	t	*P* value	Summary
Day 1	-3.000	0.3752	*P*>0.05	ns
Week 1	12.00	1.460	*P*>0.05	ns
Week 2	11.00	1.338	*P*>0.05	ns
Week 3	6.000	0.7299	*P*>0.05	ns
Week 4	5.000	0.6082	*P*>0.05	ns
Week 5	4.000	0.4866	*P*>0.05	ns
Week 6	3.000	0.3649	*P*>0.05	ns

Table B. Neuroscore mNSS value (NS) by group during study

Two-way ANOVA				
Source of Variation	% of total variation	*P*-value		
Interaction	4.37	*P*<0.0001		
Column Factor	13.74	*P*<0.0001		
NS	62.24	*P*<0.0001		
Source of Variation	*P*-value summary	Significant?		
Interaction	***	Yes		
Column Factor	***	Yes		
NS	***	Yes		
Source of Variation	Df	Sum-of-squares	Mean square	F
Interaction	4	45.2	11.3	9.674
Column Factor	2	142.1	71.04	60.82
NS	2	643.8	321.9	275.5
Residual	174	203.3	1.168	
Number of missing values	15			
Bonferroni posttests				
Vehicle vs. NFMD 1.5mg/kg				
NS	Vehicle	NFMD 1.5mg/kg	Difference	95% CI of diff.
Day 2	14.3	13.9	-0.4	-1.338 to 0.5376
Week 4	12.1	10	-2.1	-3.075 to -1.125
Week 6	11.4	9.4	-2	-2.975 to -1.025
NS	Difference	t	*P*-value	Summary
Day 2	-0.4	1.198	*P*>0.05	ns
Week 4	-2.1	6.049	*P*<0.001	***
Week 6	-2	5.761	*P*<0.001	***
Vehicle vs. NFMD 4.5mg/kg				
NS	Vehicle	NFMD 4.5mg/kg	Difference	95% CI of diff.
Day 2	14.3	13.9	-0.4	-1.348 to 0.5482
Week 4	12.1	9.1	-3	-3.975 to -2.025
Week 6	11.4	8.5	-2.9	-3.875 to -1.925
NS	Difference	t	*P*-value	Summary
Day 2	-0.4	1.185	*P*>0.05	ns
Week 4	-3	8.641	*P*<0.001	***
Week 6	-2.9	8.353	*P*<0.001	***
NFMD 1.5mg/kg vs. NFMD 4.5mg/kg				

NS	NFMD 1.5mg/kg	NFMD 4.5mg/kg	Difference	95% CI of diff.
Day 2	13.9	13.9	0	-0.9259 to 0.9259
Week 4	10	9.1	-0.9	-1.837 to 0.03658
Week 6	9.4	8.5	-0.9	-1.837 to 0.03658
NS	Difference	t	*P*-value	Summary
Day 2	0	0	*P*>0.05	ns
Week 4	-0.9	2.698	*P*=0.03	*
Week 6	-0.9	2.698	*P*=0.03	*

Table C. Change in absolute mNSS value (NS delta) by group over course of study (Day 2 - actual week)

Two-way ANOVA				
Source of Variation	% of total variation	*P*-value		
Interaction	0.01	0.9871		
Column Factor	32.51	*P*<0.0001		
NS delta	2.67	0.0322		
Source of Variation	*P*-value summary	Significant?		
Interaction	ns	No		
Column Factor	***	Yes		
NS delta	*	Yes		
Source of Variation	Df	Sum-of-squares	Mean square	F
Interaction	2	0.06632	0.03316	0.01302
Column Factor	2	145.7	72.85	28.6
NS delta	1	11.97	11.97	4.7
Residual	114	290.4	2.547	
Number of missing values	6			
Bonferroni posttests				
Vehicle vs. NFMD 1.5mg/kg				
NS delta	Vehicle	NFMD 1.5mg/kg	Difference	95% CI of diff.
Week 4	2.1	3.9	1.8	0.4990 to 3.101
Week 6	2.7	4.5	1.8	0.4990 to 3.101
NS delta	Difference	t	*P*-value	Summary
Week 4	1.8	3.511	*P*=0.003	**
Week 6	1.8	3.511	*P*=0.003	**
Vehicle vs. NFMD 4.5mg/kg				
NS delta	Vehicle	NFMD 4.5mg/kg	Difference	95% CI of diff.
Week 4	2.1	4.7	2.6	1.299 to 3.901
Week 6	2.7	5.4	2.7	1.399 to 4.001
NS delta	Difference	t	P value	Summary
Week 4	2.6	5.072	*P*<0.001	***
Week 6	2.7	5.267	*P*<0.001	***

Table D. Stepping test (ST) by group during the study

Two-way ANOVA				
Source of Variation	% of total variation	*P*-value		
Interaction	2.17	0.0069		
Column Factor	61.41	*P*<0.0001		
ST	12.64	*P*<0.0001		
Source of Variation	*P*-value summary	Significant?		
Interaction	**	Yes		
Column Factor	***	Yes		
ST	***	Yes		
Source of Variation	Df	Sum-of-squares	Mean square	F
Interaction	2	20.49	10.25	5.204
Column Factor	2	579.8	289.9	147.2
ST	1	119.4	119.4	60.63
Residual	114	224.5	1.969	
Number of missing values	6			
Bonferroni posttests				
Vehicle vs. NFMD 1.5mg/kg				
ST	Vehicle	NFMD 1.5mg/kg	Difference	95% CI of diff.
Week 4	7.6	9.9	2.3	1.156 to 3.444
Week 6	8.7	13	4.3	3.156 to 5.444
ST	Difference	t	*P*-value	Summary
Week 4	2.3	5.103	*P*<0.001	***
Week 6	4.3	9.541	*P*<0.001	***
Vehicle vs. NFMD 4.5mg/kg				
ST	Vehicle	NFMD 4.5mg/kg	Difference	95% CI of diff.
Week 4	7.6	12.6	5	3.856 to 6.144
Week 6	8.7	14.4	5.7	4.556 to 6.844
ST	Difference	t	*P*-value	Summary
Week 4	5	11.09	*P*<0.001	***
Week 6	5.7	12.65	*P*<0.001	***

Table E. Body swing difference (BSW delta) by group during the study

Two-way ANOVA				
Source of Variation	% of total variation	*P*-value		
Interaction	0.34	0.4979		
Column Factor	71.58	*P*<0.0001		
BSW delta	0.35	0.2299		
Source of Variation	*P*-value summary	Significant?		
Interaction	ns	No		
Column Factor	***	Yes		
BSW delta	ns	No		
Source of Variation	Df	Sum-of-squares	Mean square	F
Interaction	2	19.96	9.981	0.7016
Column Factor	2	4188	2094	147.2
BSW delta	1	20.72	20.72	1.457
Residual	114	1622	14.23	
Number of missing values	6			
Bonferroni posttests				
Vehicle vs. NFMD 1.5mg/kg				
BSW delta	Vehicle	NFMD 1.5mg/kg	Difference	95% CI of diff.
Week 4	15.9	6.8	-9.1	-12.17 to -6.025
Week 6	16.2	5.6	-10.6	-13.67 to -7.525
BSW delta	Difference	t	*P*-value	Summary
Week 4	-9.1	7.511	*P*<0.001	***
Week 6	-10.6	8.75	*P*<0.001	***
Vehicle vs. NFMD 4.5mg/kg				
BSW delta	Vehicle	NFMD 4.5mg/kg	Difference	95% CI of diff.
Week 4	15.9	2.7	-13.2	-16.27 to -10.13
Week 6	16.2	1.1	-15.1	-18.17 to -12.03
BSW delta	Difference	t	*P*-value	Summary
Week 4	-13.2	10.9	*P*<0.001	***
Week 6	-15.1	12.46	*P*<0.001	***

Table F. Forelimb placement (FP) test by group during the study

Two-way ANOVA				
Source of Variation	% of total variation	*P*-value		
Interaction	2.35	0.0069		
Column Factor	60.13	*P*<0.0001		
FP	11.79	*P*<0.0001		
Source of Variation	P value summary	Significant?		
Interaction	**	Yes		
Column Factor	***	Yes		
FP	***	Yes		
Source of Variation	Df	Sum-of-squares	Mean square	F
Interaction	2	27.92	13.96	5.202
Column Factor	2	714.7	357.3	133.2
FP	1	140.1	140.1	52.2
Residual	114	305.9	2.684	
Number of missing values	6			
Bonferroni posttests				
Vehicle vs. NFMD 1.5mg/kg				
FP	Vehicle	NFMD 1.5mg/kg	Difference	95% CI of diff.
Week 4	10.9	8.3	-2.6	-3.935 to -1.265
Week 6	10.1	5.5	-4.6	-5.935 to -3.265
FP	Difference	t	*P*-value	Summary
Week 4	-2.6	4.941	*P*<0.001	***
Week 6	-4.6	8.742	*P*<0.001	***
Vehicle vs. NFMD 4.5mg/kg				
FP	Vehicle	NFMD 4.5mg/kg	Difference	95% CI of diff.
Week 4	10.9	6	-4.9	-6.235 to -3.565
Week 6	10.1	3.1	-7	-8.335 to -5.665
FP	Difference	t	*P*-value	Summary
Week 4	-4.9	9.312	*P*<0.001	***
Week 6	-7	13.3	*P*<0.001	***

Table G. IL-1ß levels in injured right brain hemisphere at end of study

One-way analysis of variance					
P-value	0.6749				
P-value summary	ns				
Are means significantly different? (*P*<0.05)	No				
Number of groups	3				
F	0.4011				
R square	0.03856				
Bartlett's test for equal variances					
Bartlett's statistic (corrected)	5.646				
P-value	0.0594				
P-value summary	ns				
Do the variances differ significantly (*P*<0.05)	No				
ANOVA Table	SS	df	MS		
Treatment (between columns)	1092	2	546.1		
Residual (within columns)	27230	20	1362		
Total	28323	22			
Dunnett's Multiple Comparison Test	Mean Diff.	q	Significant? *P*<0.05?	Summary	95% CI of diff
Vehicle vs. NFMD 1.5mg/kg	14.70	0.8199	No	ns	-27.95 to 57.35
Vehicle vs. NFMD 4.5mg/kg	14.10	0.7076	No	ns	-33.30 to 61.50
