# Supplementary material for: Continuous administration of a p38α inhibitor during the subacute phase after transient ischemia-induced stroke in the rat promotes dose-dependent functional recovery accompanied by increase in brain BDNF protein level
Source: PLoS One. 2020 Dec 4;15(12):e0233073. doi: 10.1371/journal.pone.0233073 (PMC7717516; doi:10.1371/journal.pone.0233073)
Supplement: S2 Appendix — (PDF) [file pone.0233073.s005.pdf]

BDNF statistical analysis details

A) Jonckheere-Terpstra test for dose-response on BDNF levels: placebo < 1.5 mg/kg NFMD < 4.5 mg/kg NFMD

Left hemisphere

Sample count by Group
[1] 18 18 18

Mann-Whitney count by Group
[1] 200.0 241.0 208.5

sum of Mann-Whitney counts = 649.5

expected sum of Mann-Whitney counts = 486

var of sum of Mann-Whitney counts = 3969

J statistic = 2.5952380952381

P-value = 0.00472627064544695

Right hemisphere

Sample count by Group
[1] 18 18 17

Mann-Whitney count by Group
[1] 170.5 204.0 196.0

sum of Mann-Whitney counts = 570.5

expected sum of Mann-Whitney counts = 468

var of sum of Mann-Whitney counts = 3753

J statistic = 1.67314886515417

P-value = 0.0471489969497616

B) Kruskal-Wallis test with Dunn's post hoc test for multiple comparisons for analysis of BDNF levels in individual neflamapimod dose groups versus the vehicle group

Left (non-injured) hemisphere

Kruskal-Wallis test	
P value	0.0364
Exact or approximate P value?	Approximate
P value summary	*
Do the medians vary signif. (P < 0.05)?	Yes
Number of groups	3
Kruskal-Wallis statistic	6.627
Data summary	
Number of treatments (columns)	3
Number of values (total)	54

Dunn's multiple comparisons test	Mean rank diff.	Significant?	Summary	Adjusted P Value	A-?	
Vehicle vs. NFMD 1.5 mg/kg	-6.028	No	ns	0.5006	B	NFMD 1.5 mg/kg
Vehicle vs. NFMD 4.5 mg/kg	-13.47	Yes	*	0.0204	C	NFMD 4.5 mg/kg

Test details	Mean rank 1	Mean rank 2	Mean rank diff.	n1	n2	Z
Vehicle vs. NFMD 1.5 mg/kg	21.00	27.03	-6.028	18	18	1.150
Vehicle vs. NFMD 4.5 mg/kg	21.00	34.47	-13.47	18	18	2.569

Right (injured) hemisphere

Kruskal-Wallis test	
P value	0.1937
Exact or approximate P value?	Approximate
P value summary	ns
Do the medians vary signif. (P < 0.05)?	No
Number of groups	3
Kruskal-Wallis statistic	3.282
Data summary	
Number of treatments (columns)	3
Number of values (total)	53

Dunn's multiple comparisons test	Mean rank diff.	Significant?	Summary	Adjusted P Value	A-?	
Vehicle vs. NFMD 1.5 mg/kg	-1.389	No	ns	>0.9999	B	NFMD 1.5 mg/kg
Vehicle vs. NFMD 4.5 mg/kg	-8.835	No	ns	0.1813	C	NFMD 4.5 mg/kg

Test details	Mean rank 1	Mean rank 2	Mean rank diff.	n1	n2	Z
Vehicle vs. NFMD 1.5 mg/kg	23.69	25.08	-1.389	18	18	0.2699
Vehicle vs. NFMD 4.5 mg/kg	23.69	32.53	-8.835	18	17	1.692
